# Salivary anti-nuclear antibody (ANA) mirrors serum ANA in systemic lupus erythematosus

**DOI:** 10.1186/s13075-021-02694-6

**Published:** 2022-01-03

**Authors:** Ting Zhang, Yong Du, Qingqing Wu, Hao Li, Thao Nguyen, Gabriel Gidley, Valeria Duran, Daniel Goldman, Michelle Petri, Chandra Mohan

**Affiliations:** 1 Biomedical Engineering Department, University of Houston, 3517 Cullen Blvd, Room 2027, TX Houston, USA; 2 Present affiliation: The Second Affiliated Hospital, Zhejiang University School of Medicine, Hangzhou, China; 3 Division of Rheumatology, Johns Hopkins University School of Medicine, Baltimore, MD USA

**Keywords:** SLE, Saliva, ANA, Isotype

## Abstract

**Objectives:**

To assay salivary anti-nuclear antibody (ANA) and its isotypes in patients with systemic lupus erythematosus (SLE) and to investigate relevant clinical associations.

**Methods:**

Saliva samples were collected from SLE patients and assayed for salivary ANA using immunofluorescence (IF). Isotypes of salivary ANA, including IgG-ANA, IgA-ANA, and IgM-ANA, were quantified using enzyme-linked immunosorbent assay. The correlations between clinical parameters and levels of salivary ANA and isotypes were evaluated.

**Results:**

Salivary ANA IF intensities were significantly higher in SLE patients than in healthy controls, irrespective of SLE patient disease activity, and strongly correlated with serum ANA titers. Salivary ANA was detected in 67.14% of SLE patients and 10.00% of healthy controls (*p* < 0.001). Among ANA-positive samples, 80.85% exhibited a nuclear ANA pattern, and 42.55% exhibited a cytoplasmic ANA pattern. Salivary IgG-ANA, IgA-ANA, and IgM-ANA levels, as assayed by ELISA, were significantly increased in both active and less active SLE patients compared with healthy controls, and levels of each isotype were significantly correlated with serum ANA titer. Salivary IgM-ANA levels correlated with the physician global assessment (PGA), SLE disease activity index (SLEDAI), and negatively with serum C3 and C4. Salivary IgG-ANA also correlated with erythrocyte sedimentation rate (ESR), SLEDAI, and negatively with serum C3.

**Conclusion:**

Salivary ANA levels correlate with serum ANA titer, and salivary IgM-ANA and IgG-ANA correlate variably with PGA, SLEDAI, ESR and complement levels. These findings underscore the potential of using salivary ANA and ANA isotypes as surrogates for serum ANA, particularly for future point-of-care applications since saliva is easier to obtain than blood.

**Supplementary Information:**

The online version contains supplementary material available at 10.1186/s13075-021-02694-6.

## Introduction

Anti-nuclear antibody (ANA), consisting of diverse autoantibodies targeting nuclear and cytoplasmic cell components, is the serological hallmark of systemic lupus erythematosus (SLE) and is present in 95–99% of SLE patients [1–4]. Given the rarity of ANA-negative SLE patients, detection of ANA is critical to SLE diagnosis [5]. In 2019, the European League Against Rheumatism and the American College of Rheumatology defined the minimum criterion for SLE diagnosis as an ANA titer of ≥ 1:80 or at least one equivalent positive test, reflecting use of ANA as a sensitive SLE screening test [2]. ANA has also been used as an entry criterion for clinical trials of new therapeutic agents for SLE [1]. Moreover, autoantibodies play essential roles in SLE pathogenesis and have been implicated in immune complex formation and subsequent type I interferon production [1, 6, 7]. Serum ANA isotypes, including immunoglobulin G (IgG)-ANA, IgM-ANA, and IgA-ANA, have been identified in SLE patients and may have distinct roles in SLE pathogenesis [8, 9]. However, the clinical associations and potential implications of ANA isotypes in SLE pathogenesis are unclear.

The current standard for ANA testing is the immunofluorescence (IF) assay using human epithelial type-2 (HEp-2) cells, which contain various autoantigens, as substrate [4, 10]. Enzyme-linked immunosorbent assay (ELISA) has also been used for quantitative ANA measurement [11]. Although ANA is most commonly detected in serum, it is also present in various SLE patient body fluids, including pleural effusion [12], cerebrospinal fluid [13], synovial fluid [14], and urine [15]. However, ANA has not been assayed in saliva of SLE patients.

Saliva, a readily accessible specimen, has emerged as a tool for non-invasive assessment of patient health [16]. Saliva comprises several components with multiple functions and diagnostic values [17], including many plasma proteins [18]. Thus, saliva may be a useful tool for diagnosing systemic diseases. Aside from oral diseases [18], saliva biomarkers have been used to evaluate diabetes mellitus [19], acute myocardial infarction [20], and lung cancer [21]. In patients with primary Sjögren’s syndrome (pSS), an autoimmune disease with direct involvement of salivary glands, anti-SSA/B antibodies, which are included in the ANA spectrum, have been measured in saliva and assessed for diagnosis [22–24]. Given the ongoing efforts to develop point-of-care monitoring devices for rapid SLE diagnosis or home-based monitoring, this pilot study aimed to ascertain whether saliva is a viable biofluid for ANA detection and to investigate salivary ANA isotypes and their clinical correlations in SLE.

## Methods

### Study population

Saliva samples (*n* = 70) were obtained from SLE patients from the Division of Rheumatology, Johns Hopkins University (JHU) School of Medicine (Baltimore, MD, USA). Saliva samples of healthy individuals (*n* = 10) were obtained from BioIVT (New York, NY, USA) or the University of Houston (Houston, TX, USA). Informed consent was obtained from each participant, and the study was approved by the Institutional Review Boards of the JHU School of Medicine and the University of Houston. All SLE patients met the 2012 Systemic Lupus International Collaborating Clinics SLE classification criteria [5]. The following parameters were recorded: SLE disease activity index (SLEDAI), renal domains of SLEDAI (rSLEDAI), physician global assessment (PGA), complete blood count, serum creatinine, complement C3 and C4, serum ANA, and serum anti-double stranded (ds)DNA. SLE patients were categorized into two groups based on SLEDAI: (1) less active patients with SLEDAI ≤ 4 and clinical SLEDAI (omitting dsDNA and complement) ≤ 2 and (2) active SLE with SLEDAI ≥ 5 or rSLEDAI ≥ 4. Patients were not involved in the study design and conduct of this research.

### Sample collection

Whole saliva samples were collected between 7 AM and 8 AM using the Salivette® hygienic saliva collection device (Sarstedt, Nümbrecht, Germany) according to manufacturer instructions. Before sample collection, patients and healthy volunteers refrained from food or drink for ≥ 30 min, removed lipstick/balm, and rinsed their mouths without brushing or flossing teeth. The cotton swab was placed directly into the mouth, gently chewed and rolled around in the mouth for 3 min, and spat back into the tube. The capped tube was centrifuged at 1000×*g* for 2 min to yield a clear saliva sample that was collected, aliquoted, and stored at − 80 °C. The saliva volume yield ranged between 0.8 and 1.4 ml per individual.

### Indirect immunofluorescence

Saliva ANA was assayed by immunofluorescence (IF) using a commercial ANA testing kit (Catalog number: ANK120, MBL Bion, Des Plaines, IL, USA). Briefly, saliva samples were added undiluted to wells pre-coated with HEp-2 cells, incubated in a moist chamber at room temperature for 30 min, and washed with phosphate-buffered saline (PBS). Fluorescein isothiocyanate (FITC)-conjugated goat anti-human immunoglobulin, which detects the total immunoglobulin, was added to each well, and the slide was incubated for 30 min in the dark. After a PBS wash, mounting medium and coverslip were added, and the slide was examined with a confocal microscope (Nikon, Tokyo, Japan). ANA isotypes were assayed following a similar procedure using DyLight 650 conjugated goat anti-human IgG Fc antibody (1:500, Catalog Number SA5-10137, Thermo Fisher Scientific, Rockford, IL, USA), FITC-conjugated goat anti-human IgM (heavy chain) antibody (1:1000, Catalog Number A18842, Thermo Fisher Scientific), and tetramethylrhodamine isothiocyanate (TRITC)-conjugated goat anti-human IgA antibody (1:1000, Catalog Number A18786, Thermo Fisher Scientific). The ANA patterns were categorized according to the international consensus on standardized nomenclature of ANA HEp-2 cell patterns [25]. The IF staining intensities were graded independently by four trained observers (TZ, YD, QW, and HL) using a 0-4 scale based on comparison with a standard reference gallery (https://wwwn.cdc.gov/Nchs/Nhanes/1999-2000/SSANA_A.htm). The average score for each sample was calculated and recorded as the “observer score” (OS). The IF intensity of each sample was quantified using ImageJ (NIH) and recorded as the “ImageJ score” (IS).

### Elisa

To assay salivary ANA isotypes, ELISAs were performed using commercially available kits (INOVA Diagnostics, Inc., San Diego, CA, USA) containing microplates pre-coated with HEp-2 substrates. Saliva samples were diluted 1:2 in sample diluent. IgG-ANA was detected with the anti-IgG antibody provided in the kit. IgM-ANA and IgA-ANA were detected with horseradish peroxidase (HRP)-conjugated goat anti-human IgM antibody (1:20000, Catalog number 109-035-043, Jackson ImmunoResearch Laboratories, West Grove, PA, USA) and anti-human IgA antibody (1:20000, Catalog number 109-035-011, Jackson ImmunoResearch Laboratories), respectively. Standard curves for the isotypes were established with human IgG, IgA, and IgM ELISA kits (Abcam, Cambridge, MA, USA). Optical density at 450 nm was measured with a microplate reader (ELX808, BioTek Instruments, Winooski, VT, USA) and used to calculate protein concentrations.

### Statistical analyses

Data were analyzed using GraphPad Prism 7 and R. The Mann-Whitney *U* test was applied for comparisons between two groups, and chi-square test or Fisher’s exact test was used to compare percentages. The nonparametric Spearman correlation was performed for correlation analysis. The receiver operating characteristic (ROC) curve was used to determine optimal cut-off values. A two-tail *p*-value of less than 0.05 was considered significant.

## Results

### SLE patients exhibit higher salivary ANA IF intensities than healthy controls

**Fig. 1:**
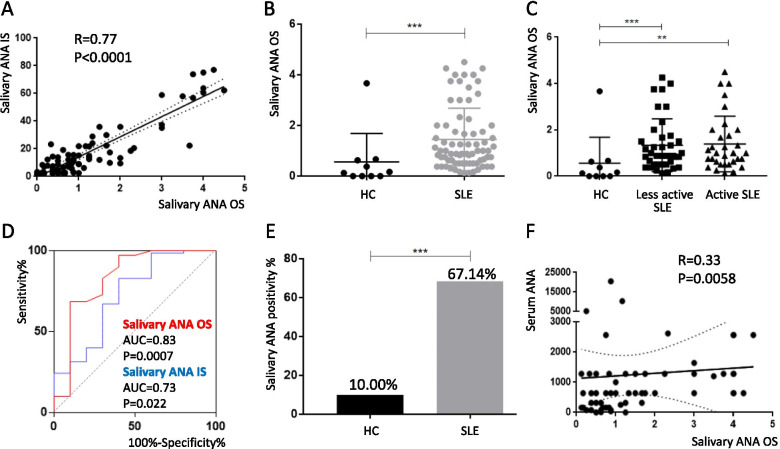
Salivary ANA positivity and IF intensity were significantly higher in SLE patients than in HC. **A** Correlation of salivary ANA observer score (OS) and ImageJ score (IS) for ANA IF intensity. Salivary ANA OS represents the average observer score of four observers. **B** ANA IF intensity was significantly higher in SLE patients (*n* = 70) than in HC (*n* = 10), **C** irrespective of the disease status of SLE patients (less active SLE, *n* = 38; active SLE, *n* = 32). **D** ROC analysis illustrating the ability of saliva ANA IF intensities to differentiate SLE patients from HC. **E** ANA positivity of SLE and HC defined by the cutoff value of OS ≥ 0.75. **F** Saliva ANA OS was significantly correlated with serum ANA titer. *P < 0.05, **P < 0.01, ***P < 0.001. ANA, anti-nuclear antibodies; IF, immunofluorescence; HC, healthy controls

Saliva samples from 70 SLE patients and 10 healthy controls were evaluated for IF intensity (Supplementary Table S1). The OS and IS values used to quantify the IF intensities of salivary ANA were highly correlated (*R* = 0.77, *p* < 0.0001) (Fig. 1A). The salivary ANA IF intensities were significantly higher in SLE patients, irrespective of disease activity, than in healthy controls (all *p* < 0.01) (Fig. 1B, C). The OS cutoff value used to differentiate samples from SLE patients and healthy individuals was determined by ROC analysis (Fig. 1D). With a salivary ANA OS cutoff value of 0.75, the corresponding salivary ANA positivity rate in healthy volunteers and patients with SLE was 10.00% and 67.14%, respectively (*p* = 0.0009) (Fig. 1E). Moreover, the salivary ANA IF intensities of SLE patients were significantly correlated with serum ANA titers (Fig. 1F).

### Salivary ANA patterns

**Fig. 2:**
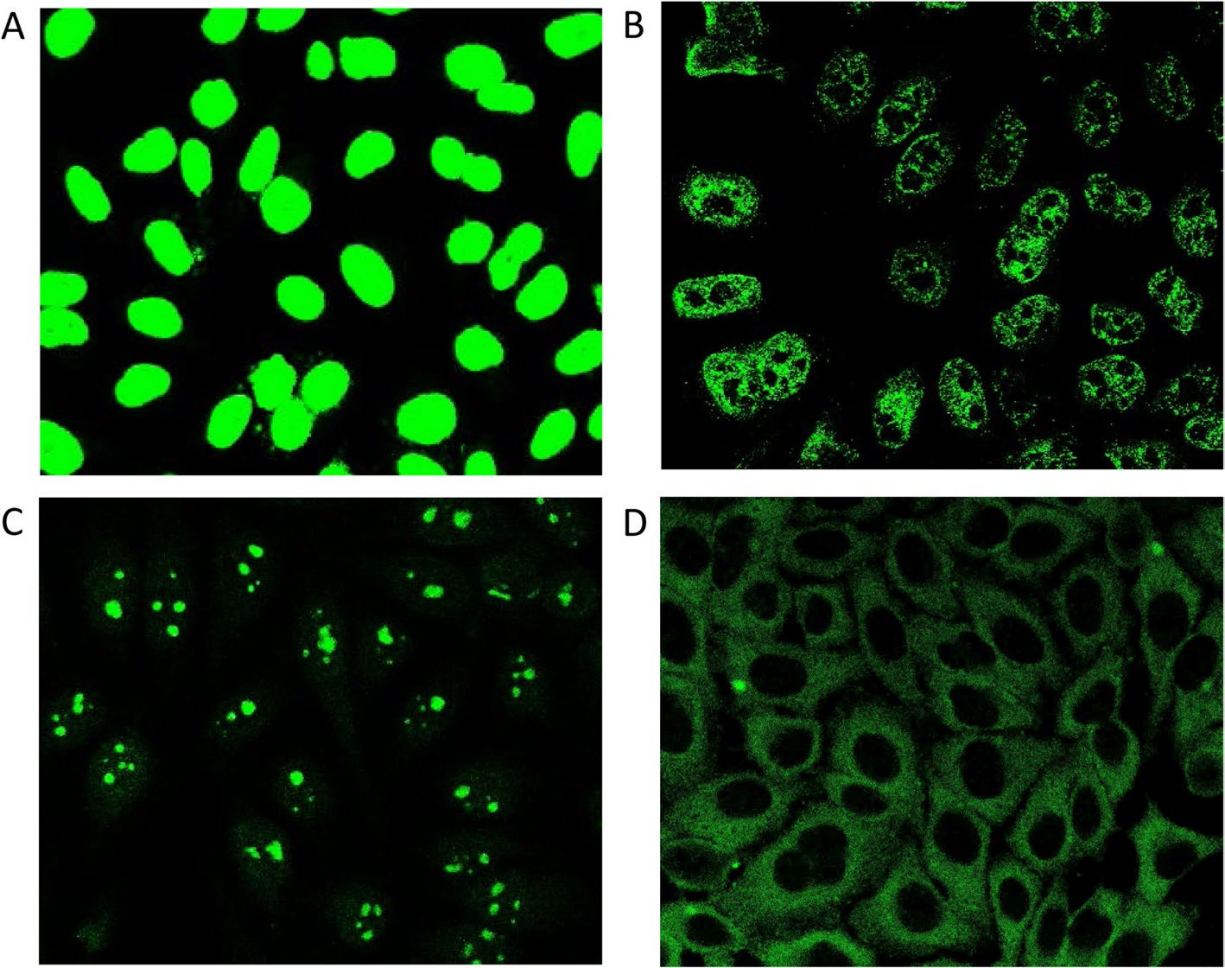
Examples of diverse salivary ANA patterns in 47 saliva-ANA-positive SLE patients using immunofluorescence. **A** Nuclear-homogeneous. **B** Nuclear-speckled. **C** Nuclear-nucleolar. **D** Cytoplasmic-speckled

Among the 47 ANA-positive SLE patients (salivary ANA OS ≥ 0.75), 38 (80.85%) exhibited a nuclear ANA pattern, and 20 (42.55%) exhibited a cytoplasmic staining pattern, with some individuals exhibiting more than one ANA staining pattern. Some samples demonstrated typical ANA sub-patterns, such as nuclear-homogeneous, nuclear-speckled, nuclear-nucleolar, and cytoplasmic speckled (Fig. 2A–D). Because further dilutions were not performed, the sub-patterns of some samples with strong IF were not confirmed, particularly samples that exhibited seemingly homogeneous nucleoplasmic staining. One healthy individual had positive salivary ANA with a nuclear pattern (not shown). ANA patterns did not correlate with disease activity as determined by SLEDAI (data not shown).

### ELISA quantification of salivary ANA isotypes

**Fig. 3:**
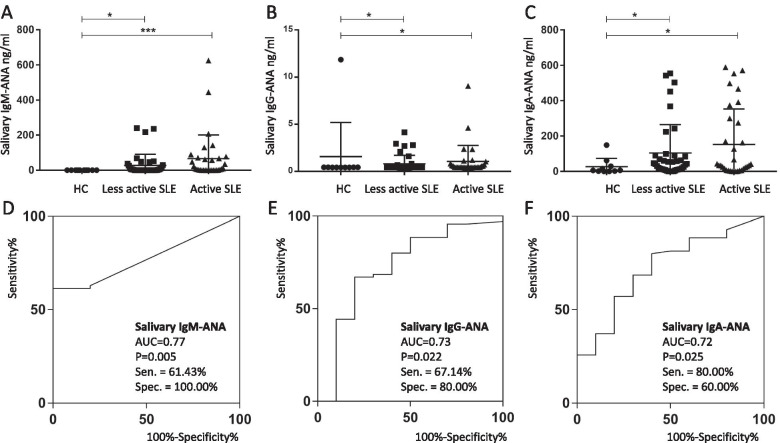
Quantification of salivary ANA isotypes in SLE, using ELISA. **A** Salivary IgM-ANA, **B** salivary IgG-ANA, and **C** IgM-ANA each was significantly higher in SLE than in HC, irrespective of SLE disease status (less active SLE, *n* = 38; active SLE, *n* = 32; HC, *n* = 10). **D–F** ROC analysis indicated that each ANA isotype discriminated SLE from HC. HC, healthy controls; Sen., sensitivity; Spec., specificity. *P < 0.05, **P < 0.01, ***P < 0.001. AUC = Area under (ROC) curve

The concentration of each salivary ANA isotype was measured by ELISA and found to be significantly higher in SLE patients than in healthy controls. Salivary ANA isotypes were significantly elevated in both active and less active SLE compared to healthy controls. ROC analysis indicated that each salivary ANA isotype significantly discriminated SLE patients from healthy individuals (Fig. 3). Whereas IgM ANA had the highest ROC AUC value (0.77), IgA ANA exhibited the highest sensitivity for SLE (at 0.80). Each ANA isotype was defined as either negative or positive based on the cutoff value resulting from ROC analysis, and combinations of ANA isotypes in discriminating SLE from healthy controls were also evaluated. However, all pairwise combinations or triple combination of ANA isotypes did not improve the discriminatory power of salivary autoantibodies (Supplementary Fig. S1).

### Correlation of salivary ANA and ANA isotypes with clinical features

**Fig. 4:**
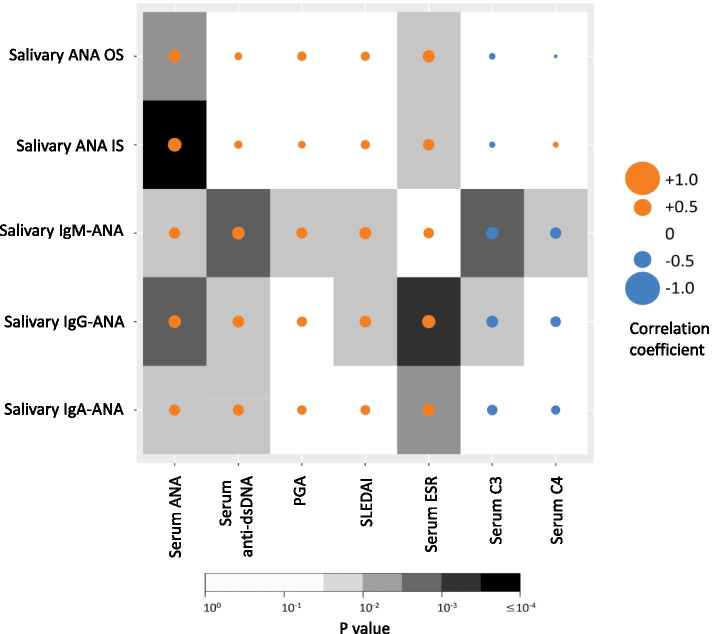
Correlation of salivary ANA and ANA isotypes with clinical parameters in SLE patients. Salivary ANA OS represents the average score from four observers. Both salivary ANA OS and IS indicate the immunofluorescent intensity of salivary ANA, whereas salivary IgM-ANA, IgG-ANA, and IgA-ANA reflect the ELISA-assayed concentrations of the ANA isotypes. The number of SLE patients assayed was 70. Analysis was done using Spearman correlation. Yellow and blue circles represent positive and negative correlation coefficient, respectively, with larger circles indicating stronger correlation. ESR, erythrocyte sedimentation rate; IS, Image J score; OS, observer score; PGA, physician global assessment; SLEDAI, systemic lupus erythematosus disease activity index

The salivary ANA OS and IS (which correlate with each other, Fig. 1A), as well as the concentrations of each ELISA-assayed salivary ANA isotype, were significantly correlated with serum ANA titers (Fig. 4). The salivary concentration of each isotype was significantly correlated with serum anti-dsDNA antibody, but a similar correlation was not observed for salivary ANA OS or IS. Salivary IgM-ANA concentration also correlated with PGA, SLEDAI, and serum C3 and C4, but did not correlate with erythrocyte sedimentation rate (ESR). Additionally, salivary IgG-ANA concentration correlated with ESR, SLEDAI, and serum C3, and IgA-ANA concentration correlated with ESR (Fig. 4). Although these correlations were significant, they were modest, with correlation coefficients ranging from 0.42 to − 0.35 (Supplementary Table S2).

**Fig. 5:**
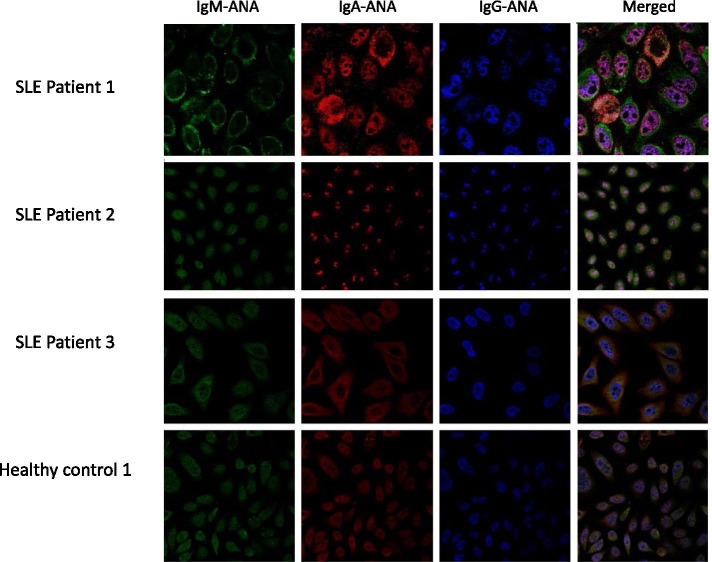
Anti-nuclear staining patterns by ANA of different isotypes in saliva of SLE patients. Salivary ANA isotypes, including IgM-ANA, IgA-ANA, and IgG-ANA from saliva of 3 SLE patients and 1 ANA+ve healthy control stained nuclei with different patterns, with some overlap between the staining patterns between the isotypes, as determined by IF. Green, IgM-ANA FITC; red, IgA-ANA TRITC; blue, IgG-ANA DyLight 650. IF, immunofluorescence

The anti-nuclear staining patterns by ANA of different isotypes in saliva of SLE patients were next examined using IF. Salivary ANA isotypes, including IgG-ANA, IgA-ANA, and IgM-ANA, from saliva of 3 SLE patients and 1 ANA+ve healthy control stained nuclei with different patterns, with some overlap between the staining patterns observed between the isotypes, as determined by IF. These limited IF studies did not reveal any evidence to suggest that salivary ANAs of different isotypes might be targeting different cellular epitopes (Fig. 5).

## Discussion

Saliva mainly originates from three paired major salivary glands and numerous minor salivary glands that generate 0.75–1.5 L of this exocrine secretion per day [18]. Saliva also contains fluids derived from oropharyngeal mucosae and gingival crevices. Plasma compounds can enter saliva by ultrafiltration through gap junctions, transudation, or selective transport through cellular membranes [16], and 20–57.1% of saliva proteins are also found in the plasma [18, 26–28]. Thus, saliva proteins can provide insight into systemic conditions and may be useful for disease monitoring or early diagnosis.

Saliva proteins perform multiple functions, including lubrication, digestion, and protection [29]. As many as 5500 proteins have been identified in saliva by proteomics [26, 30]. Immunoglobulins, including IgA, IgG, and IgM, account for 5–15% of total salivary proteins [16, 17]. However, variable salivary concentrations have been reported for each isotype [23, 31–33]. Although IgG is the dominant immunoglobulin in serum, IgA comprises > 85% of salivary immunoglobulins [16, 17]. Salivary IgA is predominantly produced as secretory IgA (sIgA) as a dimeric form by B lymphocytes located near the salivary glands [34]. Monomeric IgA derived from the serum may also be detected in whole saliva [35]. Pentameric IgM is secreted by the same mechanism as sIgA, and salivary IgG mainly derives from serum by passive diffusion [16, 17, 34]. Whether salivary ANA isotypes are generated from the same origins as immunoglobulin isotypes in saliva is unknown. Nevertheless, the elevated salivary ANA isotypes observed in SLE patients are unlikely to result from oral lesions because only one patient in our study exhibited mucosal ulcers.

The functions of different ANA isotypes also remain unclear. Few studies have investigated ANA isotypes even in the context of serum. Neither IgM-ANA nor IgG-ANA was found to be specific to any particular connective tissue disease, given that they are also detected in rheumatoid arthritis, scleroderma, and other rheumatic diseases. Serum IgG-ANA and IgM-ANA, IgG-ANA alone, and IgM-ANA alone occurred in 59.25%, 24.5%, and 4.1% of SLE patients, respectively [36]. In one study, serum IgG-ANA, IgM-ANA, and IgA-ANA were measured in SLE patients, discoid lupus erythematosus (DLE) patients, and controls with non-autoimmune diseases. IgM-ANA and IgG-ANA concentrations were significantly elevated in SLE patients compared to DLE patients and controls, and the IgA-ANA concentration was higher in SLE and DLE than in controls. However, the correlations between these isotypes and clinical or laboratory parameters were not evaluated [8]. IgM-ANA failed to produce LE cells as IgG-ANA did [9]. Whereas some IgG autoantibodies are pathogenic, IgM autoantibodies are associated with a wide spectrum of effects, ranging from injurious to protective effects depending on cellular and molecular context [37–40]. Assays using multiplexed proteome microarrays identified two IgG reactivity clusters associated with disease activity and an IgM polyreactive cluster associated with reduced disease activity in the sera of lupus patients [41].

To our knowledge, this study is the first to investigate salivary ANA and ANA isotypes in SLE patients. The IF intensity of salivary ANA and the concentration of each salivary ANA isotype significantly correlated with serum ANA levels. This result highlights the potential of using salivary ANA as a reflection of serum ANA titers, particularly in point-of-care scenarios. Interestingly, the levels of salivary ANA isotypes correlated with several clinical and conventional parameters reflective of disease activity, including SLEDAI, PGA, anti-dsDNA antibody, ESR and C3/C4 with the latter being negatively associated. Although serum ANA levels have not been considered useful serial markers of change in disease activity [42], serum ANA titers may be relevant to a patient’s immunological profile, and a positive value may signify greater disease activity [43]. Also of interest is the observation that IgA ANAs exhibited 80% sensitivity for identifying SLE, underscoring its potential utility in screening applications, particularly if this is validated in additional cohorts.

In our study, salivary ANA was detected by IF in 10% of healthy controls and 67.14% of SLE patients at the salivary ANA OS cutoff value of 0.75. Although the SLE classification criteria include ANA, as many as 20% of otherwise healthy individuals are ANA positive depending on the assay and the titer used as the cutoff for HEp2-IF [42].

ANA negativity also occurs in established SLE patients. In a cohort of 1137 SLE patients, 6.2% were ANA negative by serum IF [44]. Sera from a cohort of 103 SLE patients with historically positive ANA were assayed by three different IF kits, and the frequency of ANA negativity varied from 4.9 to 22.3% [45]. Similarly, in a cohort of 181 SLE patients with historically positive ANA and clinically active disease, five different IF kits indicated ANA negativity rates from 0.6 to 27.6% [43]. Variability in ANA negativity exists, especially in individuals with lower ANA titer [46]. In the present study, the ANA negativity in saliva may be attributed to assay and kit variability and the low abundance of salivary ANA, which restricted us from serially diluting saliva samples to determine the ANA isotype titers as regularly performed with serum samples.

One limitation of this study is the limited number of healthy control samples. Inclusion of saliva samples from control patients with other diseases, including aphthous ulcer or Behcet’s disease, and other ANA-positive autoimmune diseases, may provide additional insights. Whether the composition of salivary ANA isotypes differs between unstimulated and stimulated whole saliva is unknown. Because ANA prevalence is modestly higher in African Americans than in whites [3], validation in a larger cohort of SLE patients from multiple ethnicities is needed. The IF studies to establish the staining patterns of nuclear/cytoplasmic signals also needs to be repeated with further dilutions of the saliva, as recommended [47]. Finally, given the potential functional differences of ANA isotypes, further mechanistic studies are also warranted.

## Conclusion

Since saliva samples are easy to obtain, the main utility of salivary ANA might be in point-of-care testing and screening, although more evidence and investigations are needed before it could be applied in clinical settings. Given that salivary ANA correlated with serum ANA titer, ANA isotypes correlated with several SLE disease activity indicators, and IgA ANA had high sensitivity for SLE, salivary ANA testing warrants careful and systematic evaluation of its diagnostic and disease monitoring potential. If validated, this could pave the way towards saliva-based point-of-care ANA tests that could be performed even in a primary care setting, for rapid diagnostic evaluation of rheumatic autoimmune diseases.

## Supplementary Information

**Additional file 1: Supplementary Table S1.** Characteristics of SLE patients and healthy controls. **Supplementary Table S2**. Correlation of salivary ANA and ANA isotypes with clinical parameters. **Supplementary Figure S1.** ROC analysis indicating the discriminatory potential of combined ANA isotypes in discriminating SLE (n=70) from HC (n=10). Sen., sensitivity; Spec., specificity; AUC = Area under (ROC) curve.

## Data Availability

The data underlying this article will be shared on reasonable request to the corresponding author.

## Abbreviations

ANA: Anti-nuclear antibody
ELISA: Enzyme-linked immunosorbent assay
ESR: Erythrocyte sedimentation rate
FITC: Fluorescein isothiocyanate
HEp-2: Human epithelial type-2
HRP: Horseradish peroxidase
IF: Immunofluorescence
Ig: Immunoglobulin
IS: ImageJ score
OS: Observer score
PBS: Phosphate-buffered saline
PGA: Physician global assessment
pSS: Primary Sjögren’s syndrome
SLE: Systemic lupus erythematosus
SLEDAI: SLE disease activity index
rSLEDAI: Renal domains of SLEDAI
