# Supplementary material for: Salivary anti-nuclear antibody (ANA) mirrors serum ANA in systemic lupus erythematosus
Source: Arthritis Res Ther. 2022 Jan 3;24:3. doi: 10.1186/s13075-021-02694-6 (PMC8721993; doi:10.1186/s13075-021-02694-6)
Supplement: Supplementary file 1 — Additional file 1: Supplementary Table S1. Characteristics of SLE patients and healthy controls. Supplementary Table S2. Correlation of salivary ANA and ANA isotypes with clinical parameters. Supplementary Figure S1. ROC analysis indicating the discriminatory potential of combined ANA isotypes in discriminating SLE (n=70) from HC (n=10). Sen., sensitivity; Spec., specificity; AUC = Area under (ROC) curve. [file 13075_2021_2694_MOESM1_ESM.pdf]

Supplementary Table S1 Characteristics of SLE patients and healthy controls

Variable	HC (N=10)	All SLE (N=70)
Age (years)	39.90±7.14	42.34±13.06
Female, n (%)	10 (100.00%)	66 (94.29%)
Ethnicity, n (%)		
African-American	4 (40.00%)	28 (40.00%)
Caucasian	6 (60.00%)	33 (47.14%)
Asian	0 (0.00%)	4 (5.72%)
Other	0 (0.00%)	5 (7.14%)
Clinical Assessment		
SLEDAI		4.19±3.50
PGA		1.11±0.77
Laboratory Metrics		
WBC ($\times 10^9$/L)		5.24±2.12
HGB (g/dl)		12.42±1.51
Platelets ($\times 10^9$/L)		238.00±87.00
Serum ANA, n (%)		68 (97.14%)
C3 (mg/dl)		93.77±29.24
C4 (mg/dl)		19.94±9.09
ESR (mm/h)		33.00±29.16

SLE: systemic lupus erythematosus; HC: healthy control; SLEDAI: SLE disease activity index; PGA: physician global assessment; WBC: white blood cell; HGB: hemoglobulin; ANA: antinuclear antibody; C: complement; ESR: erythrocyte sedimentation rate.

Supplementary Table S2 Correlation of salivary ANA and ANA isotypes with clinical parameters

	Serum ANA	anti-dsDNA	PGA	SLEDAI	ESR	C3	C4
Salivary ANA OS	0.33 **	0.07	0.14	0.13	0.29 *	-0.04	-0.01
Salivary ANA IS	0.42 ***	0.09	0.07	0.14	0.26 *	-0.03	0.03
Salivary IgM-ANA	0.25 *	0.35 **	0.24 *	0.30 *	0.21	-0.35**	-0.26 *
Salivary IgG-ANA	0.34 **	0.29 *	0.19	0.27 *	0.39 **	-0.29 *	-0.21
Salivary IgA-ANA	0.24*	0.24 *	0.15	0.16	0.33 **	-0.20	-0.14

Values represent correlation coefficient. * <0.05, ** < 0.01, *** < 0.001, **** < 0.0001. Salivary ANA OS represents the average score from four observers. Both salivary ANA OS and IS indicate the immunofluorescent intensity of salivary ANA, whereas salivary IgM-ANA, IgG-ANA, and IgA-ANA reflect the ELISA-assayed concentrations of the ANA isotypes. The number of SLE patients assayed was 70. Analysis was done by Spearman correlation. ESR, erythrocyte sedimentation rate; IS, Image J score; OS, observer score; PGA, physician global assessment; SLEDAI, systemic lupus erythematosus disease activity index.

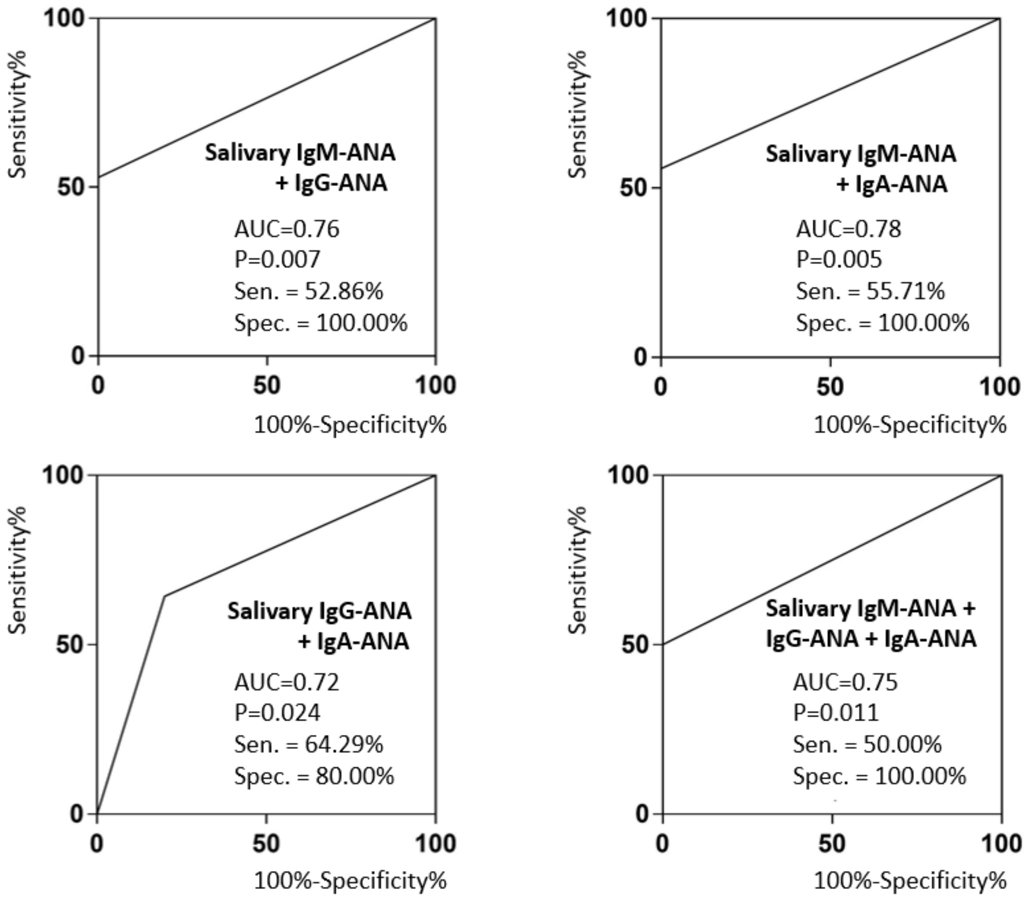

Supplementary Figure S1 ROC analysis indicating the discriminatory potential of combined ANA isotypes in discriminating SLE (n=70) from HC (n=10). Sen., sensitivity; Spec., specificity; AUC = Area under (ROC) curve.
